# Thirty-Five Years of Computerized Cognitive Assessment of Aging—Where Are We Now?

**DOI:** 10.3390/diagnostics9030114

**Published:** 2019-09-06

**Authors:** Avital Sternin, Alistair Burns, Adrian M. Owen

**Affiliations:** 1 Brain and Mind Institute, Department of Psychology, University of Western Ontario, London, ON N6A 3K7, Canada; 2 Division of Neuroscience & Experimental Psychology, Manchester Institute for Collaborative Research on Ageing, School of Social Sciences, University of Manchester, Manchester M13 9PL, UK; 3 Department of Physiology and Pharmacology, University of Western Ontario, London, ON N6A 3K7, Canada

**Keywords:** computerized cognitive assessment, aging, dementia, memory, executive function

## Abstract

Over the past 35 years, the proliferation of technology and the advent of the internet have resulted in many reliable and easy to administer batteries for assessing cognitive function. These approaches have great potential for affecting how the health care system monitors and screens for cognitive changes in the aging population. Here, we review these new technologies with a specific emphasis on what they offer over and above traditional ‘paper-and-pencil’ approaches to assessing cognitive function. Key advantages include fully automated administration and scoring, the interpretation of individual scores within the context of thousands of normative data points, the inclusion of ‘meaningful change’ and ‘validity’ indices based on these large norms, more efficient testing, increased sensitivity, and the possibility of characterising cognition in samples drawn from the general population that may contain hundreds of thousands of test scores. The relationship between these new computerized platforms and existing (and commonly used) paper-and-pencil tests is explored, with a particular emphasis on why computerized tests are particularly advantageous for assessing the cognitive changes associated with aging.

## 1. Introduction

Cognitive assessment has been of interest to psychology, cognitive neuroscience, and general medicine for more than 150 years. In the earliest reports, such as the widely-discussed case of Phineas Gage [1], cognitive ‘assessment’ was based solely on observation and subjective reports of the behavioural changes that followed a serendipitous brain injury. By the early 20th century, there had been several attempts to standardize cognitive assessments by individuals such as James Cattell [2] and Alfred Binet [3], although these were few and far between, often based on subsets of cognitive processes, and designed with specific populations in mind (e.g., children). It was not until the 1950s, 60s and 70s that the field of cognitive assessment exploded, and dozens of batteries of tests were developed, ‘normed’, and made widely available for general use (e.g., the Wechsler Adult Intelligence Scale [4], the Wechsler Memory Scale [5], the Stroop task [6,7]).

In the 1980s, a shift in emphasis occurred, as portable computers became more accessible and existing ‘paper and pencil’ cognitive assessments began to be digitized. Finally, by the turn of the century, the emergence of the world wide web made ‘internet based’ testing a reality, resulting in the creation of more reliable and efficient tests that could be taken from anywhere in the world. In parallel with the development of computerized tests for cognitive assessment, computerized brain-training games have also become popular (e.g., Lumosity). In this paper, we will only be discussing batteries designed for assessment (rather than ‘training’) purposes. Despite the proliferation of both laboratory-based and internet-based computerized cognitive assessment platforms and the many advantages they offer, these systems are still not as widely used as many of the classic paper-and-pencil batteries, particularly in older adult populations. For example, a PsychInfo search for peer-reviewed journal articles published in the 10 years between 1 July 2009 and 1 July 2019 that used the Wechsler Adult Intelligence Scale [4] and the Mini-Mental State Examination [8] in participants over the age of 65 returned 983 and 2224 studies, respectively. By comparison, when the same parameters were used to search for ‘computerized cognitive assessment’ only 364 results were returned.

The goal of this paper is to provide an overview of how both laboratory-based and internet-based cognitive assessments have evolved since the 1980s when computerized approaches were first introduced to the present day when they routinely make use of small, ultra-portable technologies such as cell phones and tablets (e.g., iPads). We will focus our discussion on how these assessments are being applied to detect and track dementia. Key differences between these new computerized platforms and existing (and commonly used) paper-and-pencil tests will be discussed, with a particular emphasis on why computerized tests are particularly advantageous for assessing the cognitive changes associated with aging.

## 2. Computerized Cognitive Assessment—Historically

The computerization of cognitive assessment tools began in the 1980s with the development of personal computers. Although initial digitization efforts mainly focused on the straight conversion of paper-and-pencil tests to computerized formats, new methods of assessment soon began to be developed that capitalized on emerging technologies (such as touchscreens, response pads, computer mice, etc.). These new methods, when used alongside computers to collect data, led to the creation of tests that were more efficient at assessing an individual’s abilities than their paper-and-pencil equivalents. For example, computerized tests are able to measure response latencies with millisecond accuracy and record and report on many aspects of performance simultaneously. Computers can calculate scores and modify test difficulty on the fly, as well as automate instructions, practice questions, and administration of the tests across large groups of people—something that is not so easy for a human test administrator to accomplish. Moreover, because test difficulty can be adjusted on-the-fly, assessments can be shorter and therefore less frustrating, or exhausting, for impaired individuals. In addition, predefined criteria can dictate the maximum number of successes or failures that each individual is exposed to, such that the subjective experience of being tested is equivalent across participants. The reporting of scores also becomes easier and more accurate because their interpretation can be made entirely objectively based on calculated statistics using information gleaned from large normative datasets.

Some of these advantages lead to greater test sensitivity [9] and as such, computerized cognitive assessments are valuable for investigating changes that may not be detected using conventional methods. This makes them ideal for assessing and following subtle cognitive changes in aging over the long term and increases the possibility that emerging mild cognitive impairments will be detected as early as possible [10].

An early example of a set of computerized cognitive tests was the Cambridge Neuropsychological Test Automated Battery (CANTAB). CANTAB was originally designed for the neuropsychological assessment of neurodegenerative diseases and was the first touch-screen based, comprehensive, computerized cognitive battery. CANTAB was standardized in nearly 800 older adult participants [11], and early studies indicated that specific tests, or combinations of tests, were sensitive to deficits and progressive decline in both Alzheimer’s disease and Parkinson’s disease [12,13,14,15,16]. Specific tests from the CANTAB battery also appear to be able to predict the development of dementia in preclinical populations, while also differentiating between different disorders such as Alzheimer’s disease and Frontotemporal dementia [10,17,18]. This early example of a computerized neuropsychological battery paved the way for others, designed to assess similar, or different, types of cognitive function and dysfunction (e.g., Cambridge Brain Sciences [19], Automatic Neuropsychological Assessment Metrics [20], Computerized Neuropsychological Test Battery [21], Touch Panel-Type Dementia Assessment Scale [22]).

Although the broad body of literature that has accumulated over the last 35 years indicates that computerized tests are adept at detecting and monitoring cognitive decline in neurodegenerative disorders, little consensus exists about which are the most effective and suitable for this task. Two recent reviews described 17 such batteries as being suitable for use in aging populations (see [23,24] for tables illustrating these batteries in detail). The consensus across both reviews was that, although broadly valid for testing aging populations, many of these batteries had serious shortcomings. For example, many batteries relied on normative data from small samples sizes or samples that lacked data specific to older adults. Ultimately, both reviews suggested that how useful any given battery was must be assessed on a case-by-case basis and that no one test, or battery of tests, could be singled out as being the most reliable for screening and monitoring cognitive impairment in the elderly. Without doubt, this general lack of consensus about computerized cognitive tests has contributed to their slow adoption into health care systems. Clinicians are rightly hesitant to adopt any new platform for screening or monitoring patients when normative population data are lacking [25], and this issue needs to be urgently resolved. The obvious way to accomplish this is to greatly increase the number of participants who have completed any given computerized test or battery, and generate norms based on these large databases that can be used to assess the performance of groups or individuals with known, or suspected, clinical disorders. For practical and economic reasons, this is not feasible when assessments need to be taken by a trained administrator in a laboratory testing environment. However, with the advent of the internet, mass ‘self-administration’ of computerized cognitive tests has become a reality, opening up many new and transformative opportunities in this domain.

## 3. Cognitive Assessment in the Internet Age

The internet and the proliferation of portable computers into every aspect of our lives (e.g., phones, TVs, tablets), has created many new opportunities, and challenges, for computerized cognitive assessment. For example, by making cognitive assessments available online, a much larger number of participants can be reached than would be possible when the tests are administered on paper and/or in a laboratory setting. With increasing numbers, demographic variables such as age, geographical location and socioeconomic status can also be fed into each assessment, and on-the-fly comparisons with large normative databases can be used to provide ‘personalized’ results that take these factors into account.

One example of such an online tool is the Cambridge Brain Sciences (CBS) platform. The tests in this battery are largely based on well validated neuropsychological tasks but have been adapted and designed to capitalize on the numerous advantages that internet and computer-based testing can offer. The CBS battery has been used to conduct several large-scale population-based studies involving tens of thousands of participants from all over the world [19,26], as well as more than 300 bespoke scientific studies (e.g., [27,28,29]). As testament to the ‘power of the internet’, in total, more than 8 million tests have been taken, and normative data from 75,000 healthy participants are available, including approximately 5000 adults over the age of 65.

Having access to such a large number of datapoints also makes it possible to investigate how demographic factors affect cognition in a way and on a scale that was never before feasible, shedding new light on the interplay between biology and environmental factors and their effects on cognitive function. For example, in one recent study of 45,000 individuals, the CBS battery was used to examine the influence of factors like gender differences, anxiety, depression, substance abuse, and socio-economic status on cognitive function, as well as how they interact during the aging process to uniquely affect different aspects of performance [30].

Other computerized assessment batteries that have been used in older adult populations include the Automatic Neuropsychological Assessment Metrics [20], Computerized Neuropsychological Test Battery [21], and the Touch Panel-Type Dementia Assessment Scale [22]. Each of these batteries consists of a series of tests designed to measure various aspects of cognitive functioning such as processing speed, memory retention, and working memory using tasks based on command following, object recognition, logical reasoning, mathematical processing, and symbol-digit coding.

### 3.1. Meaningful Change

When normative databases include tens of thousands of participants, it becomes possible to compute indices that are simply not possible with smaller (e.g., lab-based) data samples. Estimates of ‘meaningful’ or ‘reliable’ change are one such example that has particular relevance for monitoring cognitive decline or improvement on an individual basis. Estimates of meaningful or reliable change compare the difference in an individual’s performance on a task between two time points (e.g., between a patient’s current assessment results and previous baseline results) to the variability in repeated measurements that would occur in the absence of a meaningful change. The latter is estimated from a sample of healthy control subjects, and the larger that sample is, the better. Gathering data from a large number of individuals via online testing allows for a database of thousands of normative data points [19,31]. The meaningful change index used by the CBS platform, for example, uses the test-retest reliability and the standard deviation of scores (measured in the control sample) of each task to describe the range of possible differences that could occur with repeated task completion. If an individual’s change in performance from one time point to another is much larger than expected by chance (i.e., larger than the fluctuations seen in the control sample), then one can conclude that there was a meaningful change. This may be crucial for evaluating a single aging patient and deciding whether or not a change in performance from one assessment to the next is ‘meaningful’ or simply a reflection of the day to day fluctuations that are characteristic of healthy cognitive functioning. The above method of calculating a meaningful change score is one example of how computerized cognitive testing can be used to monitor cognitive changes over time. Other methods that, for example, investigate the longitudinal measurement invariance can also be used [32,33] to determine whether the scores from a single metric collected over time are stable or changing in a meaningful way.

The increased size of the normative database to which individual scores are compared is one way in which modern internet-based assessment tools are able to address an issue raised by Zygouris and Tsolaki [24]; that is, physicians rarely have time to wade through the complicated data output of computerized testing batteries to interpret their meaning. When the meaning of test results can be determined through automated statistical algorithms that interrogate a large normative database, the task of interpreting test results is offloaded to the battery itself (something that is clearly not possible with traditional pencil-and-paper methods). When a meaningful change is detected, caregivers or health care providers can be alerted ‘automatically’ so that more in-depth testing can be initiated to assess the individual’s cognitive status. This has relevance in home care, assisted living facilities, and in hospital settings for reducing the administrative burden of monitoring cognitive changes, while also increasing the sensitivity of testing to catch important changes early enough to be appropriately addressed. This in turn, increases the likelihood that physicians will be amenable to adopting these methods for monitoring and screening aging individuals because the logistic and economic overheads are low. In addition, the immediate delivery, objectivity, and interpretation of scores makes them straightforward for non-experts to understand and increases the probability that these methods will be adopted into the broader health care system because any health care provider or family member can, in principle, monitor an aging patient’s cognitive changes over time, the effect of drugs, or even cognitive changes post-surgically [34].

### 3.2. Validity of At-Home Testing

**Figure 1:**
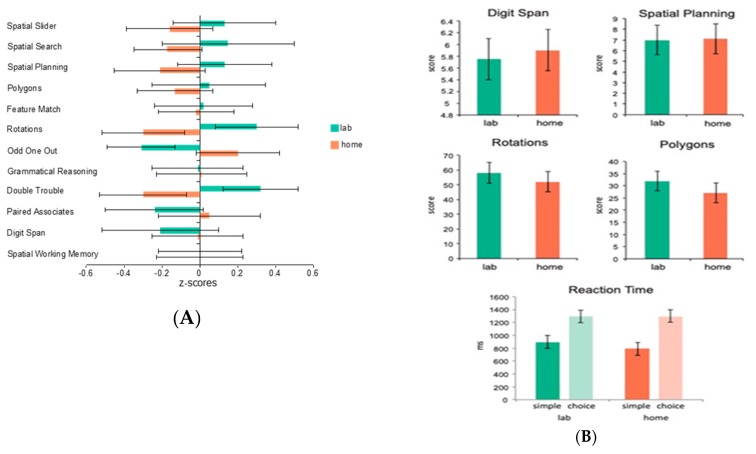
(**A**) average standardized scores on the 12 Cambridge Brain Sciences (CBS) tasks taken at home and in the lab by 19 healthy young adult controls. The results showed no significant effect of at home versus in laboratory testing (*F =* 1.71, *p* = 0.2); (**B**) average raw scores on 4 CBS tasks as well as simple and choice reaction time tasks taken at home and in the lab by 27 patients with Parkinson’s Disease. Again, there was no significant effect of at home versus in-lab testing (*p* > 0.1) and the tasks showed reliable correlations across the two testing environments (*p* < 0.05).

As we have implied above, one of the main advantages of internet-based testing is that it can be conducted at home (or theoretically, anywhere), as long as a computer with an internet connection is available. One of the obvious questions, however, is its validity in comparison to in-lab testing. To assess this question, we had 19 healthy young adult control participants complete the full CBS battery (12 tests) both while unsupervised at home and while supervised in the laboratory (test order was counterbalanced across participants). The mean standardized scores for each of the tests showed no significant effect of at home versus in laboratory testing (*F =* 1.71, *p* = 0.2) and the tasks showed reliable correlations within participants across the two testing environments (*p* < 0.05) (See Figure 1A). A follow-up study explored whether the stability in scores across testing environments was applicable to patient groups as well as healthy controls. A total of 27 participants with Parkinson’s disease were assessed on 4 of the 12 CBS tests at home and in-lab as well as tests of simple and choice reaction time similar to the ones included in the CANTAB battery (the order of tasks was counterbalanced across participants). Again, there was no significant effect of at home versus in-lab testing (*p* > 0.1), and the tasks showed reliable correlations across the two testing environments (*p* < 0.05) (See Figure 1B). Moreover, the results of the simple and choice reaction time tasks demonstrated that response time measures could be collected accurately over the internet, regardless of the testing platform used. Together, the results of these two studies indicate that computerized tests taken unsupervised at home produce results no different than those taken in a laboratory, both in healthy controls and in a patient population.

**Figure 2:**
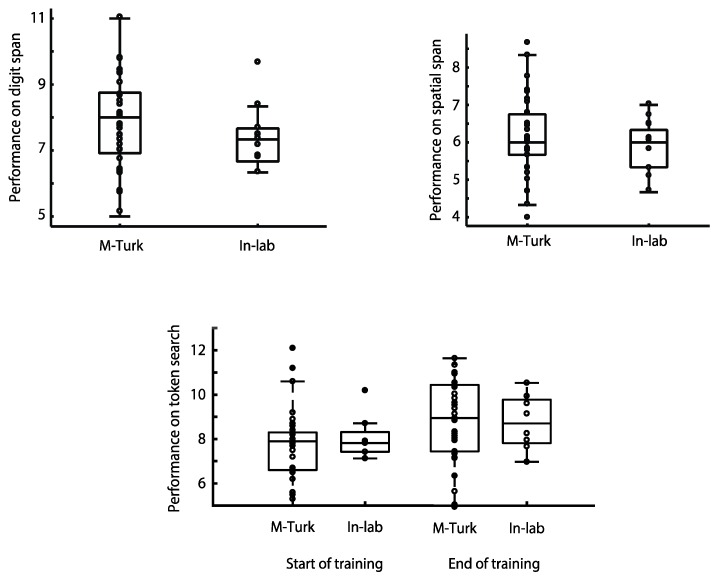
Average scores on 3 CBS tasks (Digit Span, Spatial Span and Token Search), taken at home and in the lab by more than 100 young adult controls. The results showed no significant effect of at home versus in laboratory testing [35]. In the case of Token Search (lower panel), the overlap in performance for participants tested at home using Amazon’s MTurk and those tested in the laboratory persisted even after several weeks of intensive training on the task [35].

In a third recently published study examining the relationship between unsupervised cognitive testing ‘at home’ and supervised lab-based assessment, the performance of more than 100 participants was compared on three of the CBS tests, Digit Span, Spatial Span and Token Search [35] There were no significant differences in performance between those participants who completed the tests online via Amazon’s MTurk platform and those who completed the testing supervised within the laboratory (Figure 2). In the case of the Token Search test, this was even true after extensive training on the task over several weeks [35].

Another advantage of internet-based testing and large-scale normative databases is that it is relatively straightforward to calculate indicators of ‘validity’ on-the-fly which can then be used to ‘flag’ when testing has not been completed properly, or according to the instructions. By analyzing thousands of data points, a set of parameters can be defined that must be met for a score on a test to be considered valid. Including a simple and easy-to-read marker on a score report that conveys whether performance on a task is within reasonable bounds increases the usability and confidence in the test by health care providers.

Finally, there are other mechanisms that can be used to ensure reliable data are collected when tasks are self-administered at home in online settings. For example, interactive learning tutorials can guide participants through practice trials and objectively determine when an individual has understood task instructions before beginning a testing session. Such practice trials increase the validity of the tests, particularly when they are taken for the first time.

## 4. Online Testing vs. Existing Alternatives

The ability to quickly and accurately assess changes in cognitive functioning on a regular basis has implications for quality of life, level of independence, and degree of care in the aging adult population. Currently, assessments like the Mini-Mental Status Exam (MMSE) [8] and the Montreal Cognitive Assessment (MoCA) [36] are used by health care providers to monitor cognitive changes and screen for deficits. Although these tests are useful because they are short and easy to administer, there are some downsides to using these paper-and-pencil based methods of assessment. For example, they are not adaptive to an individual’s ability level, which can lead to frustration in patients with deficits or unnecessary redundancy in individuals who are clearly completely unimpaired. In addition, the questions are not randomly generated with each administration (so opportunities for retesting are reduced). Third, these tests must be administered by a trained individual, which introduces testing bias and takes time and resources away from other health care duties. Fourth, rather than detecting fine grained changes in cognition, these paper-pencil tests assign patients to very broad categories (impaired or unimpaired)—binary classification of this sort is highly susceptible to error through day to day fluctuations in normal cognitive functioning. Finally, the cutoff scores used in these tests may not be appropriate for aging populations [37,38,39] and result in larger numbers of patients being labeled as ‘impaired’ than perhaps is necessary.

Several recent studies have investigated whether short computerized assessments can effectively monitor cognitive changes over time and better differentiate between older adult populations with differing abilities than the most widely used paper-and-pencil alternatives. When 45 older adults recruited from a geriatric psychiatry outpatient clinic were tested on five computerized tests from the CBS battery, results showed that some of these tests provided more information about each individual’s cognitive abilities than the standard MoCA when administered on its own [40]. The addition of scores from just two of the computerized tests (total testing time of 6 min) to a MoCA, better sorted participants into impaired or unimpaired categories. Specifically, 81% of those patients who were classified as being borderline (between ‘impaired’ and ‘not impaired’) based on their MoCA scores alone were reclassified as one or the other when scores from two computerized tests were introduced. Additionally, this study demonstrated that some computerized tests provide more information than others when used in this context. That is to say, two of the five tests employed were not at all useful in classifying borderline patients and the fifth test was too difficult for the older adults to understand and complete.

To follow-up this study, we recently investigated whether other tests in the CBS battery, beyond the five used by Brenkel et al. [40], could provide more information about older adults’ cognitive abilities, as well as whether traditional tests like the MoCA or the MMSE could be replaced entirely by an online computerized assessment battery.

A total of 52 older adults (average age = 81 years, 62–97 years) were asked to complete the 12 online tests from the CBS battery in random order. Each task was presented on a touchscreen tablet computer and was preceded by instructions and practice trials. Afterwards, the MoCA (version 7.1 English) and MMSE were administered in interview format, always by the same person (AS). Possibly because of the location of the retirement homes from which participants were recruited, the sample was highly educated. All but one earned high school diplomas, 24 earned postsecondary degrees, and 16 earned postgraduate degrees. Two participants did not complete all 12 tasks due to fatigue and loss of interest; thus 50 participants’ scores were analysed. MoCA scores ranged from 12–30 (mean = 24.6) and MMSE scores ranged from 16–30 (mean = 27.7; see Supplementary Figure S1).

**Figure 3:**
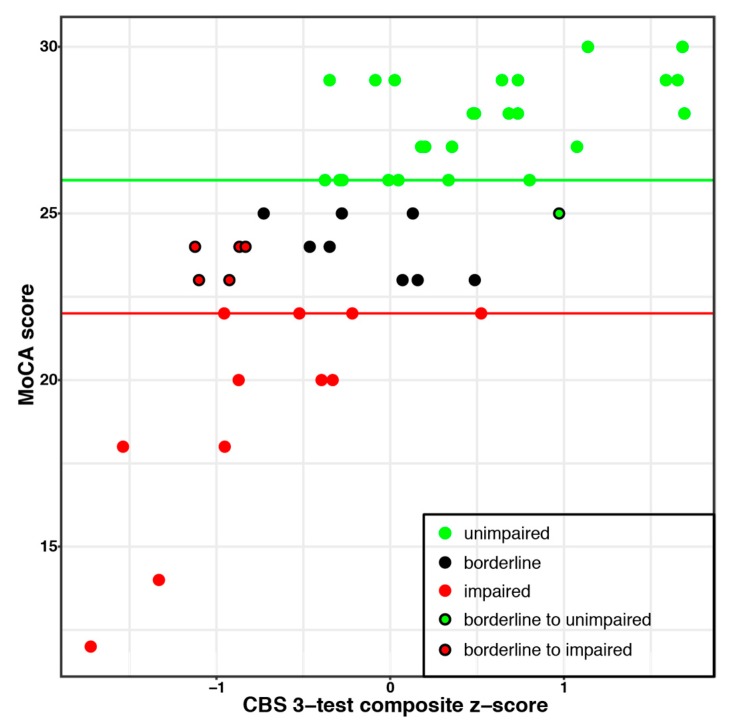
The CBS composite score was highly correlated with Montreal Cognitive Assessment (MoCA) scores and better differentiated impaired and unimpaired individuals. The border colour of each datapoint indicates the categorization of individuals based on MoCA scores alone. The fill colour indicates to which group borderline participants are categorized when the composite score of 3 CBS tests is used.

Participant scores were split into three categories based on the results of the MoCA test (See Figure 3): unimpaired (*n* = 25; MoCA score ≥ 26), borderline cognitive impairment (*n* = 14; MoCA score 23–25), and impaired (*n* = 12; MoCA score ≤ 22), based on thresholds from previous literature (e.g., [36,37,38]). Each participant in the borderline MoCA group was then reclassified to either the impaired or unimpaired groups based on their CBS test scores. A ceiling effect precluded such an analysis for the MMSE results.

Using the MoCA score alone, 72% of participants were classified as impaired or unimpaired. The addition of a single CBS task (Spatial Planning) improved this classification to 92% of the participants. This was not simply because Spatial Planning was the most difficult test, as the equally difficult Spatial Span test left 5 participants in the borderline group. Test difficulty was determined from an unrelated study with scores from 327 participants age 71–80 (see Supplementary Figure S2).

A second analysis using a step-wise multiple regression indicated that MoCA scores were best predicted by two additional CBS tests: Odd One Out and Feature Match (*R*^2^ = 0.65). Age did not significantly predict any variance over and above these tests. Alone, age predicted 22% of the variance in MoCA scores (*R*^2^ = 0.22). Another step-wise multiple regression showed that MMSE scores were best predicted by Feature Match and Grammatical Reasoning (*R*^2^ = 0.38). Again, age did not explain a significant amount of variance over and above the task scores. Alone, age predicted 8% (*R*^2^ = 0.08) of the variance in MMSE scores.

A third regression showed that level of education did not explain a significant amount of variance in MMSE or MoCA scores, although this may be due to overall high educational levels and the ceiling effect seen in MMSE scores (see Supplementary Figure S1).

Scores on the three CBS tasks identified in the two analyses (Feature Match, Odd One Out, Spatial Planning) were then combined to create a composite score. The composite score was highly correlated with MoCA scores and was better than the MoCA alone at differentiating impaired from unimpaired participants (84% versus 72% for the MoCA on its own; see Figure 3).

The results discussed above illustrate the potential that short computerized tests have as screening tools for efficiently monitoring cognitive changes over time. This study also suggests that minimal computer literacy is required when using a touchscreen tablet as technical limitations did not preclude individuals from participating. Another potential use for computerized testing is as a replacement for, or supplement to, neuropsychological assessments that are used for the diagnosis of various brain disorders. In one recent foray into this area, the relationship between a 30 min computerized testing battery and a standard 2–3 h neuropsychological assessment [41] was explored in 134 healthy adults (mean age was 47 years). Although the computerized testing battery could not account for significant variance in the assessments of verbal abilities (e.g., WASI Vocabulary subtest, Word List Generation), it did account for 61% of the variance in the remainder of the traditional neuropsychological battery. The results confirmed that a 30 min internet-based assessment of attention, memory, and executive functioning was comparable to a standard 2–3 h neuropsychological test battery and may even have some diagnostic capabilities.

In the sections above, we have sought to illustrate our arguments with just a few examples of how cognitive changes in older adults can be effectively monitored using self-administered, internet-based computerized testing batteries. Although further validation is required in some cases, there is already good reason to believe that a shift towards internet-based computerized cognitive testing in health care may be warranted.

## 5. Neural Validation

A key aspect to cognitive assessment is validating the areas of the brain that are involved in the cognitive functions in question. This has long been the domain of neuropsychologists who use results from neurally validated assessments to triangulate brain function from behavioural assessment results. Historically, cognitive assessments were validated using brain lesion studies, but the rise of imaging technologies has made the neural validation of newly developed cognitive assessments accessible and easier to complete.

Coincidentally, the computerization of cognitive assessments has grown alongside this increase in the availability of imaging tools. These parallel timelines have resulted in many examples of computerized cognitive tasks that have been validated from the get-go with neural information gleaned from neuroimaging studies [19,42]. Importantly, however, these imaging studies have underscored the fact that there is rarely a one-to-one mapping between cognitive functions and the brain areas, or networks, that underpin them. One approach to this issue is to examine the complex statistical relationships between performance on any one cognitive task (or group of tasks) and changes in brain activity to reveal how one is related to the other. In order to do this most effectively, large amounts of data need to be included because of the natural variance in cognitive performance (and brain activity) across tests and across individuals. In the age of computerized internet testing and so-called ‘big data’, this problem becomes much easier to solve. Thus, the sheer amount of data that can be collected allows statistical tests to be performed that were simply not possible when data were collected by hand. For example, Hampshire et al. [19] collected data on the 12 CBS tasks from 45,000 participants. These data were then subjected to a factor analysis, and 3 discrete factors relating to overall cognitive performance were identified. Each one of these factors represents an independent cognitive function that is best described by a combination of performance on multiple tests, something that no single test can assess, and were labeled as encapsulating aspects of short-term memory, reasoning, and verbal abilities, respectively. This technique allows an individual’s performance to be compared to a very large normative database in terms of these descriptive factors rather than performance on a single test.

As an example of how this might be applied to a question related directly to aging, Wild et al. [31] recently used this same approach to investigate how sleeping patterns affect cognitive function across the lifespan in a global sample of more than 10,000 people. Using the same analysis of factor structure employed previously by Hampshire et al. [19], the results showed that the relationship between sleep and short-term memory, reasoning and verbal factors was invariant with respect to age, overturning the widely-held notion that the optimal amount of sleep is different in older age groups. Indeed, sleep-related impairments in these three aspects of cognition were shown to affect all ages equally, despite the fact that, as expected, older people tended to sleep less [31]. Put simply, the amount of sleep that resulted in optimal cognitive performance (7–8 h), and the impact of deviating from this amount, was the same for everyone—regardless of age. Somewhat counter-intuitively, this implies that older adults who slept more or less than the optimal amount were impacted no more than younger adults who had non-optimal sleep. If sleep is especially important for staving off dementia and age-related cognitive decline [43], then one might predict that a lack of sleep (or too much sleep) would be associated with more pronounced cognitive impairment in the elderly than in younger adults. Nonetheless, given that 7–8 h of sleep was associated with optimal cognition for all ages and that increasing age was associated with less sleep, the results suggest that older populations in general would likely benefit from more sleep.

Additionally, the neural networks responsible for cognitive factors that are derived from analysing data from multiple tests across large samples of participants can be assessed. For example, Hampshire et al. [19] described the neural correlates of each factor in a group of 16 healthy young participants who completed the testing battery in an fMRI scanner. The short-term memory factor was related to activation in the insula/frontal operculum, the superior frontal sulcus, and the ventral portion of the anterior cingulate cortex and pre-supplementary motor areas. The reasoning factor was related to activation in the inferior frontal sulcus, the inferior parietal cortex, and the dorsal portion of the anterior cingulate and pre-supplementary motor areas. The verbal factor was related to activation in the left inferior frontal gyrus and the bilateral temporal lobes. These data indicate that identifying the neural correlates of cognitive functions is possible with a very large database of participants allowing for complex statistical tests to be performed to interrogate their complex inter-relationships. Computerized assessments are particularly suited to the task of collecting thousands of datapoints and combined with imaging data can provide valuable insights into how a brain injury or neural degeneration as a result of aging affects the brain networks responsible for complex cognitive functions.

## 6. Conclusions

Computerized cognitive assessments have come a long way in the past 35 years. The proliferation of technology has resulted in reliable and easy to administer batteries that have great potential for affecting how the health care system monitors and screens for cognitive changes in the aging population. Importantly, modern computerized and internet-based cognitive tasks have been designed to capitalize on the many advantages that computers can offer to create more efficient and accurate assessments than existing paper-and-pencil options. One of the key advantages is the way in which these tasks are scored and interpreted. Computerized tests can use statistical measures to interpret one individual’s score within the context of thousands of normative data points and provide an objective interpretation of that individual’s performance ‘on-the-fly’. This shift moves away from the traditional intuition-based approach that more typically required a highly trained individual to interpret a constellation of test scores.

The objective nature of computerized test scores has implications for the adoption of these test batteries into health care because they do not need to be administered or interpreted by a highly trained individual. These batteries can be used by physicians, family members, or other front-line health care workers to monitor for subtle changes in cognition. Catching these changes and flagging them for a more thorough follow-up with the appropriate health-care professional helps to improve quality of life in patients with declining cognitive abilities as well as moves the responsibility of monitoring cognitive changes from a few highly trained individuals to a large number of front-line health-care providers. In short, self-administered online cognitive testing batteries have the potential to help close the dementia diagnosis gap without adding undue burden to the existing health care system.

## Supplementary Materials

The following are available online at https://www.mdpi.com/2075-4418/9/3/114/s1.
