# Supplementary material for: Thirty-Five Years of Computerized Cognitive Assessment of Aging—Where Are We Now?
Source: Diagnostics (Basel). 2019 Sep 6;9(3):114. doi: 10.3390/diagnostics9030114 (PMC6787729; doi:10.3390/diagnostics9030114)
Supplement: Supplementary file 1 [file diagnostics-09-00114-s001.pdf]

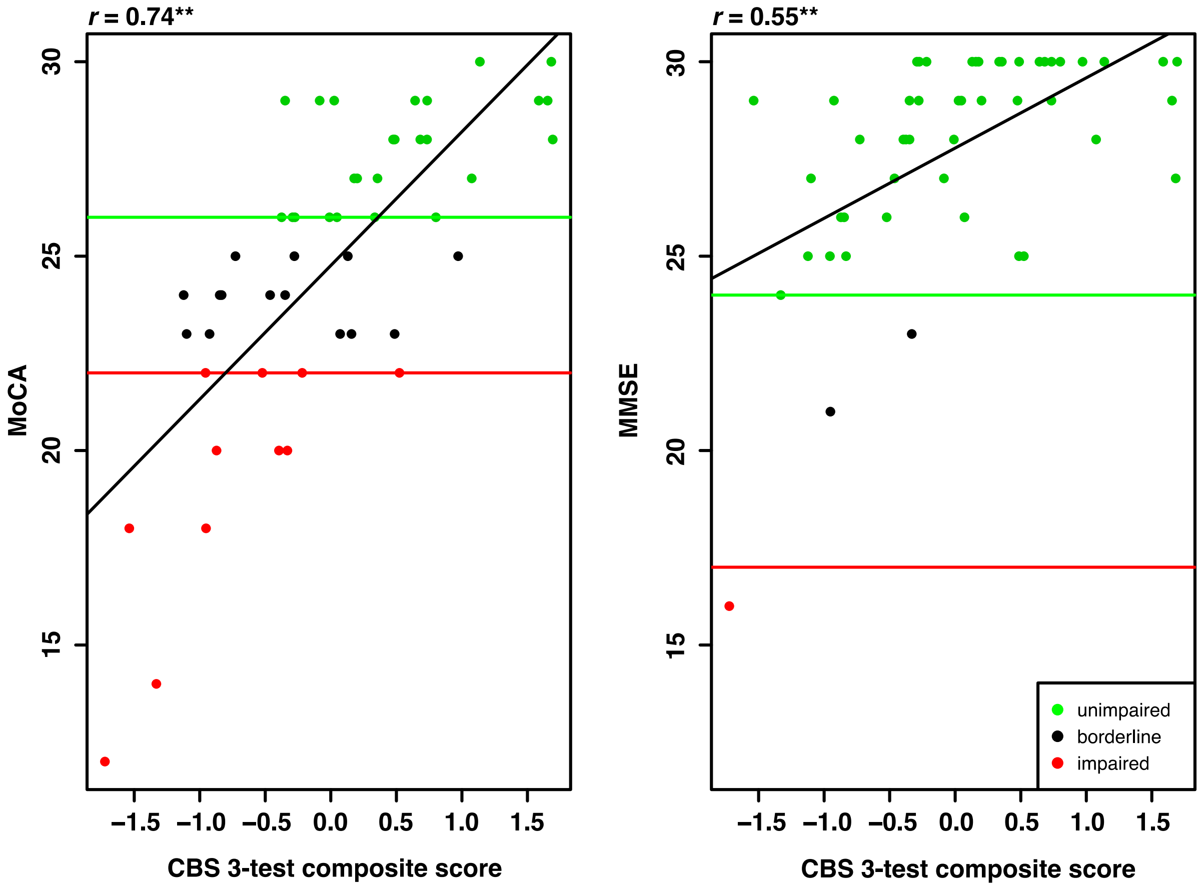

Figure S1. MoCA and MMSE scores are plotted here with horizontal lines indicating the thresholds used to differentiate the three groups. MoCA scores were differentiated using the method described in this paper. MMSE scores were differentiated using the severity method as explained in the published MMSE scoring document[8]. Correlations between the tests and composite scores were significant at $p<0.001$.

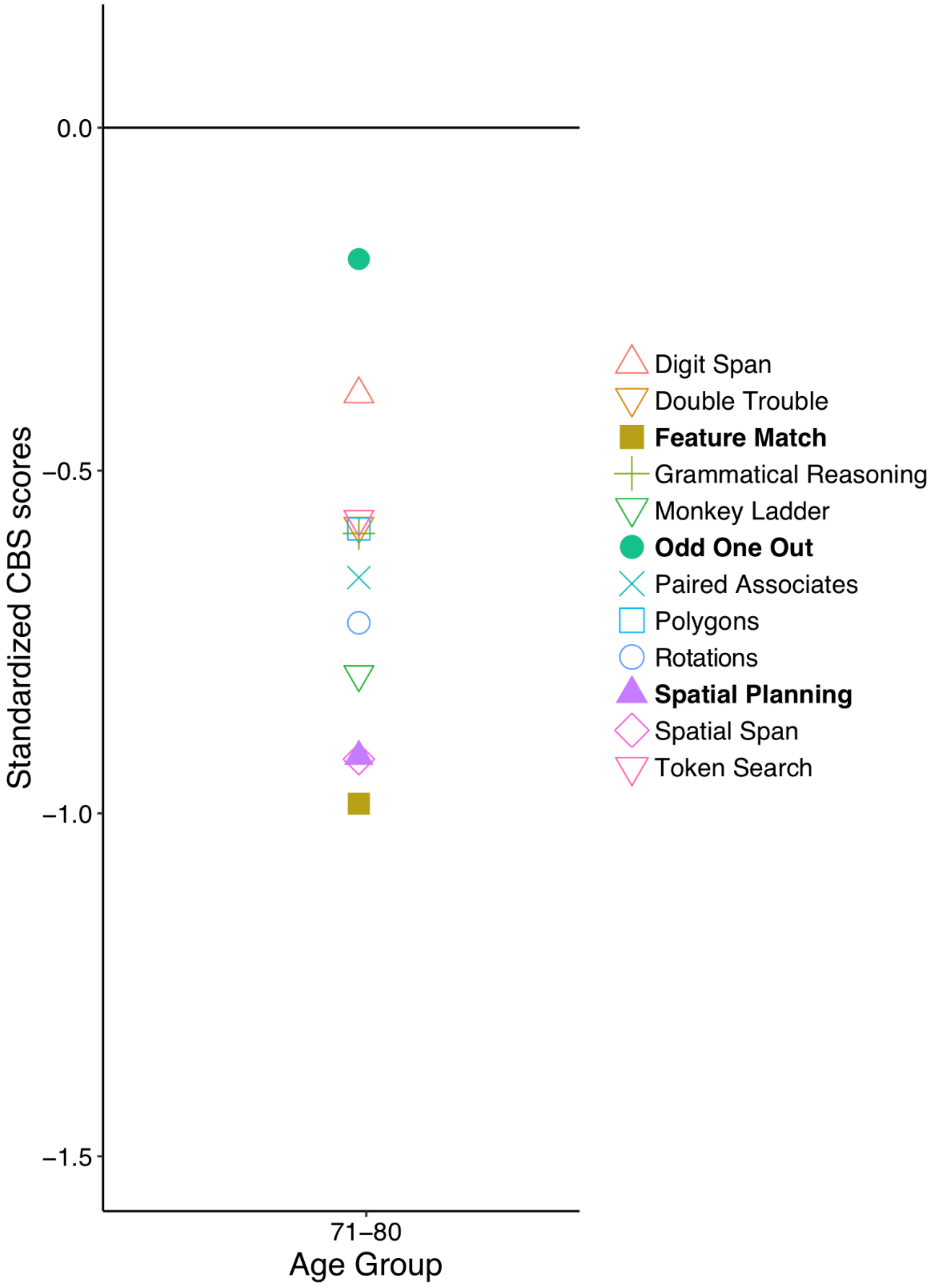

Figure S2. As part of a large online study 327 participants (age 71-80 years) completed all 12 CBS tasks. The standardized scores are presented in this figure and show relative task difficulty. The three tests in bold (Feature Match, Odd One Out, Spatial Planning) are those identified by the analyses in the current paper. As described in the main text, task difficulty cannot account for the effects observed.
